# Clinical, pathological and dermoscopic phenotype of *MITF* p.E318K carrier cutaneous melanoma patients

**DOI:** 10.1186/s12967-020-02253-8

**Published:** 2020-02-13

**Authors:** Giulia Ciccarese, Bruna Dalmasso, William Bruno, Paola Queirolo, Lorenza Pastorino, Virginia Andreotti, Francesco Spagnolo, Enrica Tanda, Giovanni Ponti, Cesare Massone, Francesco Drago, Aurora Parodi, Giovanni Ghigliotti, Maria Antonietta Pizzichetta, Paola Ghiorzo, Italian Melanoma Intergroup (I.M.I.), Bruna Dalmasso, William Bruno, Lorenza Pastorino, Virginia Andreotti, Paola Queirolo, Francesco Spagnolo, Enrica Tanda, Maria Antonietta Pizzichetta, Paola Ghiorzo

**Affiliations:** 1 IRCCS Ospedale Policlinico San Martino, Genetics of Rare Cancers, Genoa, Italy; 2 Department of Internal Medicine and Medical Specialties (DiMI), University of Genoa, Genoa, Italy; 3 IRCCS Ospedale Policlinico San Martino, Medical Oncology 2, Genoa, Italy; 4 Department of Diagnostic and Clinical Medicine and Public Health, Division of Clinical Pathology, University of Modena and Reggio Emilia, Modena, Italy; 5 Galliera Hospital, Dermatology Unit, Genoa, Italy; 6 Department of Health Sciences (Di.S.Sal.), University of Genoa, Genoa, Italy; 7 IRCCS Ospedale Policlinico San Martino, Section of Dermatology, Genoa, Italy; 8 Dermatology Clinic - National Cancer Institute, Medical Oncology and Preventive Oncology Aviano, University of Trieste, Aviano, Italy

**Keywords:** Melanocyte Inducing Transcription Factor, E318K, Cutaneous melanoma, Renal cell carcinoma, Nevi, Dysplastic nevi, Dermoscopy, Germline variant, Susceptibility, Cancer genetics

## Abstract

**Background:**

The p.E318K variant of the Melanocyte Inducing Transcription Factor (*MITF*) has been implicated in genetic predisposition to melanoma as an intermediate penetrance allele. However, the impact of this variant on clinico-phenotypic, as well as on dermoscopic patterns features of affected patients is not entirely defined. The purpose of our study was to assess the association between the p.E318K germline variant and clinic-phenotypical features of *MITF*+ compared to non-carriers (*MITF*−), including dermoscopic findings of melanomas and dysplastic nevi.

**Methods:**

we retrospectively analyzed a consecutive series of 1386 patients recruited between 2000 and 2017 who underwent genetic testing for *CDKN2A*, *CDK4*, *MC1R* and *MITF* germline variants in our laboratory for diagnostic/research purposes. The patients were probands of melanoma-prone families and apparently sporadic single or multiple primary melanoma patients. For all, we collected clinical, pathological information and dermoscopic images of the histopathologically diagnosed melanomas and dysplastic nevi, when available.

**Results:**

After excluding patients positive for *CDKN2A*/*CDK4* pathogenic variants and those affected by non-cutaneous melanomas, our study cohort comprised 984 cutaneous melanoma patients, 22 *MITF*+ and 962 *MITF*−. *MITF*+ were more likely to develop dysplastic nevi and multiple primary melanomas. Nodular melanoma was more common in *MITF*+ patients (32% compared to 19% in *MITF*−). *MITF*+ patients showed more frequently dysplastic nevi and melanomas with uncommon dermoscopic patterns (unspecific), as opposed to *MITF*− patients, whose most prevalent pattern was the multicomponent.

**Conclusions:**

*MITF*+ patients tend to develop melanomas and dysplastic nevi with histopathological features, frequency and dermoscopic patterns often different from those prevalent in *MITF*− patients. Our results emphasize the importance of melanoma prevention programs for *MITF*+ patients, including dermatologic surveillance with digital follow-up.

## Background

Malignant melanoma is a potentially lethal tumor resulting from the malignant transformation of melanocytes. A recent meta-analysis showed that 70.9% of melanomas likely arises de novo from melanocytes located in previously normal skin, mucous membranes or other sites (eye, inner ear, gastrointestinal system) and 29.1% arises from melanocytes in pre-existing lesions (nevi or dysplastic nevi). Ultraviolet light, especially indoor tanning, exposure is a known carcinogen clearly correlated with melanoma.

The worldwide incidence of melanoma is increasing and it is estimated to further increase mainly due to the lengthening of the human lifetime and the aging of population: currently, the lifetime risk of developing melanoma is 1 in 63 in the United States and in other Western countries.

Along with lifestyle, genetic risk factors are significant conditions contributing to melanoma development [1, 2].

A number of novel candidate melanoma predisposition genes, in addition to the well know high penetrance genes such as Cyclin Dependent Kinase Inhibitor 2A (*CDKN2A*), Cyclin-Dependent Kinase 4 (*CDK4*) and low-penetrance genes such as Melanocortin 1 Receptor (*MC1R*) have been uncovered in the last few years. These include genes involved in DNA replication (telomere maintenance) or repair, such as Telomere Reverse Transcriptase (*TERT*), Protection of Telomeres 1 (*POT1*), Adrenocortical Dysplasia (*ACD*), Telomeric Repeat-Binding Factor-2 (*TERF2*) Interacting Protein (*TERF2IP*), and Breast Cancer Gene 1 (*BRCA1*)-Associated Protein 1 (*BAP1*). Moreover, the p.E318K variant of the Melanocyte Inducing Transcription Factor (*MITF*) gene has recently been implicated in cancer predisposition [3–5]. The groups of Bertolotto [6] and Yokoyama [7] independently identified the p.E318K variant and categorized *MITF* as an intermediate penetrance melanoma susceptibility gene. Indeed, patients carrier of this germline variant have a more than fivefold increased risk of developing melanoma (both familial and sporadic), renal cell carcinoma (RCC) or both cancers than non-carriers. The p.E318K variant has also been associated with increased nevus count, non-blue eye color and developing of multiple primary melanoma [7]. *MITF* is the ‘master regulator’ of differentiation, survival and proliferation of normal melanocytes and is critical in controlling proliferation, migration and invasion of melanoma cells [8]. It has been demonstrated that *MITF* acts not only as a master transcription factor, involved in cell cycle regulation, but also as a transcriptional repressor [9]. The p.E318K variant alters the SUMOylation of *MITF* thus impairing *MITF* inhibitory activity [6, 7]. More specifically, in normal cells under normoxia, the small ubiquitin-like modifier (SUMO) proteins, bind *MITF* decreasing the transcription of the hypoxia inducible factor 1 A (HIF1A). In addition, HIF1A is hydroxylated for subsequent proteasome mediated degradation of the cells. Under hypoxia, SUMOs are released, allowing the transcription of HIF1A and anaerobic metabolism or glycolysis. The p.E318K variant of *MITF* in melanoma and renal carcinoma cells severely impaired SUMOylation of *MITF*, resulting in an increased transcription of HIF1A and other genes compared to wild-type *MITF*. Even under normoxic conditions, the p.E318K variant allows cancer cells to activate a pseudohypoxic response or aerobic glycolysis and this “Warburg effect” predispose to cancer progression and metastasis [10].

Due to the above-mentioned links with melanoma and kidney cancer susceptibility, current research is focusing on the relationship between p.E318K and the clinico-phenotypic features of individuals carrying this variant [9, 11–16]. However, current literature on dermoscopic features of nevi and melanomas in these patients is still limited, and predisposition to non-melanoma cancers according to *MITF* germline status needs to be further investigated [11, 14, 16].

The aim of the present work was to retrospectively study genotype–phenotype correlations in melanoma patients carrier of the p. E318K *MITF* germline variant (*MITF*+), compared with non-carrier melanoma patients (*MITF*−). Among the analyzed phenotypic features, we included dermoscopic findings of histopathologically diagnosed dysplastic nevi (DN) and cutaneous melanomas in *MITF*+ and *MITF*−.

## Methods

### Patients characteristics

Between 2000 and 2017 we recruited a consecutive series of 1386 patients. This cohort included probands of melanoma-prone families and apparently sporadic patients diagnosed with multiple primary melanomas who underwent genetic testing for diagnostic or research purposes, as well as apparently sporadic patients with melanoma, tested for research purposes only. All patients, except one case already characterized for germline status, were subjected to genetic testing for *CDKN2A*, *CDK4*, *MC1R* and *MITF* germline variants in our laboratory. For all patients, we collected and stored clinical and pathological information. In addition, when available, we collected dermoscopic images of the histopathologically diagnosed DN and cutaneous melanomas.

Indeed, the histopathological diagnosis of DN is based on the presence of both of the two major criteria (proliferation of atypical melanocytes extending beyond the dermal component; atypical melanocytes arranged in a lentiginous/epithelioid-cell pattern) and at least two minor criteria (lamellar/eosinophilic fibrosis; neovascularization; inflammatory response; fusion of rete ridges) [17].

For 667 of the patients included in this study, molecular and, partly, clinical information have been previously described [13].

### Collection of clinical, pathological and dermoscopic data

Clinical information were collected through a questionnaire, administered by a trained interviewer, and included phenotype and personal/family history of melanomas and other tumors, as previously described [18, 19]. Either clinical records or local cancer registry data were used to collect pathological information, including tumor histological type and staging according to the American Joint Committee on Cancer (AJCC)’s TNM staging system [20, 21]. For both *MITF*+ and *MITF*− patients, the following phenotypical and clinico-pathological features were studied: phototype, freckles, hair and eye color, total number of nevi with diameter > 2 mm, number of histopathologically diagnosed DN and number of histopathologically diagnosed cutaneous melanomas. For first diagnosed melanomas, we also gathered information on age at diagnosis, anatomical site, histotype, Breslow thickness (mm), sentinel lymph node and stage.

Dermoscopic images of lesions clinically suggestive of melanomas were collected through the FotoFinder dermoscope Medicam 1000 (FotoFinder Systems GmbH, Bavaria, Germany) during dermatologic visits performed for screening (first visit) or for follow-up at the Dermatologic Clinic of the San Martino Hospital (Genoa, Italy) and at the Dermatologic outpatient clinic, Division of Oncology, Centro di Riferimento Oncologico, Aviano National Cancer Institute (Aviano, Italy).

The analysis of global dermoscopic pattern was retrospectively performed on all available dermoscopic images of non-acral lesions according to the dermoscopic classification of acquired melanocytic nevi, as follows: reticular, globular, homogenous, multicomponent, reticular-globular, reticular-homogenous, globular-homogenous and unspecific pattern. This latter was defined as a pattern lacking specific features related to a melanocytic or non melanocytic lesions. Conversely, the following dermoscopic patterns were considered to assess acral melanocytic lesions: parallel furrow, parallel ridge, lattice-like, fibrillary [22, 23]. All the dermoscopic images were evaluated by a panel of three independent observers; the dermoscopic features were scored based on the agreement of two observer (G.C. and F.D.) and in case of disagreement between the two observers, a third observer (M.A.P.) was consulted. The evaluation of the dermoscopic criteria was made when 3/3 or 2/3 observers agreed.

### Molecular analysis

All patients provided a blood sample from which we extracted genomic DNA. Purified DNA samples were then amplified by conventional polymerase chain reaction (PCR) and analyzed by Sanger sequencing to assess the germline status of *CDKN2A*, *CDK4*, *MC1R* and *MITF*. Samples processing and analysis were performed as previously described [24, 25].

### Patients selection

From the melanoma cohort, we excluded the patients lacking information on germline status and tumor stage, as well as those with non-cutaneous melanoma (ocular and mucosal melanomas). Moreover, to avoid confounding effects by *CDKN2A* and *CDK4,* patients with concurrent *CDKN2A* and *CDK4* pathogenic variants were also excluded from this study. Subsequently, we gathered *MITF*+ and *MITF*− patients into two separate study groups. All patients signed a written informed consent according to local ethics committee approved protocol prior to enrolment in the study.

### Statistical analysis

To assess the difference of a numerical variable between the two study groups (age at diagnosis, Breslow thickness, number of melanomas diagnosed, total number of nevi, number of dysplastic nevi) we used the Mann–Whitney U test.

To assess the association between *MITF*+ germline status and a categorical variable (hair-eye color, familial status, sentinel node status, familiarity for pancreatic and kidney cancer, site and histotype of first melanoma, dermoscopic pattern of DN and melanomas grouped together, *MC1R* germline status, histopathologically diagnosed melanomas and DN analyzed as a dichotomous variable), we used the Fisher’s exact test.

Kruskal–Wallis test was used to analyze the association between *MITF* germline status and an ordinal variable (phototype, freckles, tumor stage and number of nevi grouped in three categories).

## Results

**Fig. 1:**
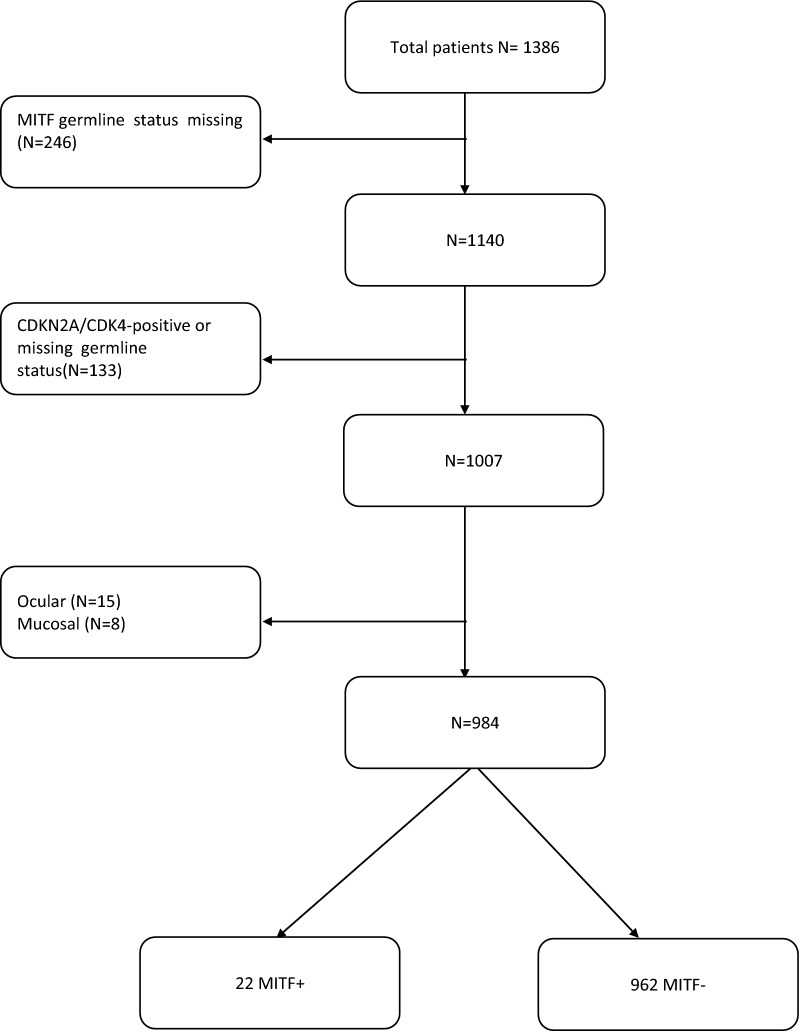
Patients selection workflow

After excluding 246 patients with missing information on *MITF* mutational status, 133 patients either positive for *CDKN2A*/*CDK4* pathogenic variants or with missing information on *CDKN2A*/*CDK4* germline status, and 23 patients affected by ocular or mucosal melanomas, our study cohort comprised 984 cutaneous melanoma patients, 22 *MITF*+ and 962 *MITF*− (Fig. 1). Of the 22 *MITF*+ patients, 5 had a positive family history of melanoma, whereas the remaining 17 were apparently sporadic cases. Overall, 6 patients, all apparently sporadic cases, developed multiple melanomas. Even though the overall prevalence of the *MITF* p.E318K variant was 2.2% (22 of 984), *MITF* p.E318K was more common among multiple primary melanoma (MPM) patients (5% compared to 2% in single melanoma patients). All MPM *MITF*+ patients were sporadic, whereas among single primary melanoma (SPM) patients *MITF* p.E318K rate was similar in familial and sporadic subgroups.

The distribution of *MC1R* variants did not significantly differ between the two study groups (p = 0.45, Table 1). In the *MITF*+ group, two patients had amelanotic/hypomelanotic melanomas; both patients carried one red-hair-color (RHC) *MC1R* variant (R169W, R142H).

**Table 1:** Clinical, pathological and molecular characteristics of the study groups

	N		*MITF*+ N (%)	*MITF*− N (%)	OR^a^	Lower CI	Upper CI	p-value
Phototype	927	I	0 (0)	52 (6)				0.27
		II	15 (75)	478 (53)				
		III	5 (25)	353 (39)				
		IV	0 (0)	24 (3)				
Freckles	349	None	8 (4)	77 (23)				0.37
		Rare	4 (2)	126 (38)				
		Few	7 (35)	89 (27)				
		Many	1 (5)	37 (11)				
Hair color	932	Albino	0 (0)	1 (0)				0.06
		Red	1 (5)	49 (5)				
		Blond	9 (43)	201 (22)				
		Blond_red	0 (0)	9 (1)				
		Brown	7 (33)	565 (62)				
		Black	4 (19)	86 (9)				
Eye color	931	Light blue	7 (37)	214 (23)				0.50
		Blue	1 (5)	38 (4)				
		Green	2 (11)	98 (11)				
		Grey	0 (0)	21 (2)				
		Light brown	1 (5)	206 (23)				
		Dark brown	8 (42)	324 (36)				
		Black	0 (0)	5 (1)				
		Hazel	0 (0)	6 (1)				
Number of nevi	492	< 10	3 (17)	200 (42)				*0.04*
		10–50	10 (55)	185 (39)				
		> 50	5 (28)	89 (19)				
Histologically diagnosed Dysplastic nevi	866	Median (IQR)	0 (0–1)	0 (0–0)				*< 0.001*
		0	10 (50)	769 (91)	9.93	3.59	27.53	*< 0.001*
		1+	10 (50)	77 (9)				
Familial	984	Spo	17 (77)	833 (87)	1.9	0.54	5.48	0.21
		Fam	5 (23)	129 (13)				
Age at first melanoma	858	Median (IQR)	44 (32.25–58.75)	49 (38.99–61.71)				0.20
N. of melanomas removed	983	Median (IQR)	1 (1–1.75)	1 (1–1)				*0.02*
		1	16 (73)	855 (89)	3.02	0.95	8.35	*0.03*
		2+	6 (27)	106 (11)				
Breslow mm	930	Median (IQR)	1 (0.6–2.025)	1 (0.35–1.765)				0.22
Sentinel node	337	Neg	6 (27)	266 (28)	1.41	0.14	8.1	0.65
		Pos	2 (9)	63 (7)				
Stage	771	IS	2 (17)	122 (16)				0.65
		I	8 (67)	473 (62)				
		II	2 (17)	75 (10)				
		III	0 (0)	56 (7)				
		IV	0 (0)	33 (4)				
Pancreatic cancer in family	972	No	19 (86)	901 (94)	2.90	0.53	10.35	0.11
		Yes	3 (14)	49 (5)				
Kidney cancer in family	971	No	18 (82)	910 (95)	5.17	1.21	16.74	*0.01*
		Yes	4 (18)	39 (5)				
Site of first melanoma	943	Head and neck	1 (5)	74 (8)				0.27
		Trunk	8 (40)	464 (50)				
		Arms	6 (30)	125 (14)				
		Legs	5 (25)	260 (28)				
Histotype of first melanoma	722	Acral	2 (9)	11 (2)				*0.04*
		Lentigo maligna	0 (0)	36 (5)				
		Nodular	7 (32)	132 (19)				
		SSM	13 (59)	454 (64)				
		Other	0 (0)	67 (10)				
MC1R	576	–/–	6 (30)	165 (30)				0.45
		r/–	4 (20)	158 (28)				
		r/r	2 (10)	24 (4)				
		R/–	6 (30)	111 (21)				
		R/r	1 (5)	74 (13)				
		R/R	1 (5)	24 (4)				

Significant p-values are italicized

N = number of patients, % = percentage of patients, OR = odds ratio, lower CI = lower confidence interval limit, upper CI = upper confidence interval limit, IQR = inter-quartile range, Spo = sporadic, Fam = familial, Neg = negative, Pos = positive, SSM = superficial spreading melanoma, IS = in situ melanoma, R = MC1R red hair color variant, r = MC1R non-red hair color variant

^a^Odds that the outcome occurs in the *MITF*+ group compared to the odds of the outcome occurring in the *MITF*− group

### Clinico-pathological features

The two study groups displayed significant differences with regards to: total number of nevi, number of histopathologically diagnosed DN and melanomas, histotype of first melanoma and family history of kidney cancer (Table 1). More specifically, *MITF*+ patients had, in median, a higher total number of nevi compared to *MITF*− patients: 28% of *MITF*+ patients had more than 50 nevi, compared to 19% of *MITF*− patients (p = 0.04).

The number of melanomas removed was higher in *MITF*+ compared to *MITF*− negative patients: 27% of the *MITF*+ patients have removed more than 2 melanomas versus 11% of the *MITF*− patients (p = 0.03).

Patients with at least one histologically diagnosed DN were more frequent in the *MITF*+ group, (50% vs. 10% in the *MITF*− group, p < 0.001) with a higher median DN removal compared to *MITF*− (median = 0.5, IQR = 0–1 and median = 0, IQR = 0–0, respectively; p < 0.001).

Concerning the histotype of first melanoma, *MITF*+ patients showed a higher rate of nodular melanomas than *MITF*− patients (32% and 19%, respectively, p = 0.04).

Patients with MPM were more frequently *MITF*+ (27% compared to 11% of *MITF*− patients, p = 0.03). A positive family history for kidney cancer was more frequent among *MITF*+ patients (18% versus 5% of *MITF*− patients; p = 0.01).

We also compared phenotypical features between the *MITF*+ and *MITF*− patients, and no significant differences were found as regards phototype, hair and eyes color, freckles, age at first melanoma diagnosis, anatomical site, Breslow thickness, sentinel lymph node, stage of first melanoma and family history of melanoma or pancreatic cancer (Table 1).

### Dermoscopic features

The dermoscopic patterns of 23 lesions (including DN and melanomas) belonging to four *MITF*+ patients were compared with those of 47 lesions (DN and melanomas) belonging to 37 *MITF*− patients (Table 2).

**Table 2:** Dermoscopic patterns of *MITF *+ and *MITF*− dysplastic nevi and cutaneous melanomas

Dermoscopic pattern	*MITF*+		*MITF*−		p-value
	N	%	N	%	
Structureless	6	26	4	8	*< 0.001*
Reticular	1	4	1	2	
Globular	0	0	3	6	
Homogeneous	2	9	0	0	
Globular-homogenous	5	22	2	5	
Reticular-homogenous	6	26	3	6	
Reticular-globular	0	0	12	26	
Multicomponent	2	9	22	47	
Parallel ridges (or other patterns typical of acral melanoma)	1	4	0	0	
Total	23	100	47	100	

Significant p-values are italicized

N = number of dysplastic nevi/melanomas; % = percentage of dysplastic nevi/melanomas

When dermoscopically evaluating only melanomas, nine lesions belonging to three *MITF*+ were compared with those of 23 lesions belonging to 22 *MITF*− (Table 3).

**Table 3:** Dermoscopic patterns of *MITF*+ and *MITF*− cutaneous melanomas

Dermoscopic pattern	*MITF*+		*MITF*−	
	N	%	N	%
Structureless	4	45	3	13
Reticular	1	11	0	0
Globular	0	0	2	9
Homogeneous	0	0	0	0
Globular-homogenous	1	11	1	4
Reticular-homogenous	0	0	0	0
Reticular-globular	0	0	2	9
Multicomponent	2	22	15	65
Parallel ridges (or other patterns typical of acral melanoma)	1	11	0	0
Total	9	100	23	100

N = number of melanomas; % = percentage of melanomas

Of the 23 dermoscopic images from the four *MITF*+ patients, seven melanomas (four with structureless, two with multicomponent and one with globular-homogenous pattern) and ten DN (two with homogenous, four with reticular-homogenous and four with globular-homogenous pattern) belonged to one single patients. This patient actually developed 10 melanomas, only 7 of which had dermoscopic images.

**Fig. 2:**
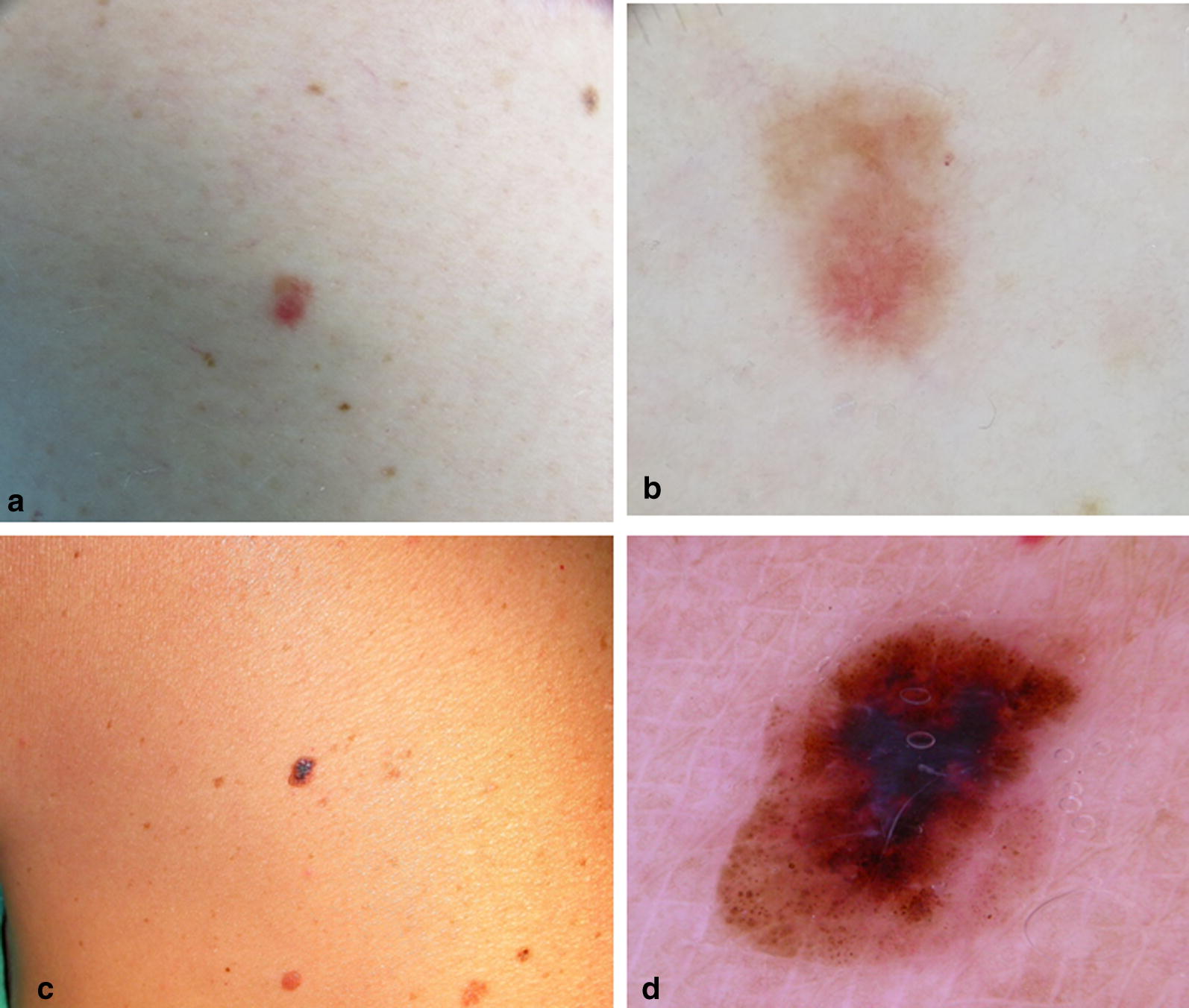
**a** Clinical and **b** dermoscopic images of a superficial spreading melanoma of the right thigh with an unspecific dermoscopic pattern in a *MITF*+ patient; this patient also carries one RHC variant (R142H) of MC1R that could be responsible for the hypomelanotic aspect of this lesion; **c** clinical and **d** dermoscopic images of a superficial spreading melanoma of the left shoulder with a multicomponent pattern in a *MITF*− patient

When we analyzed the global patterns of DN and melanomas, grouped together as a single variable, the unspecific, globular-homogenous and reticular-homogenous patterns were more frequent in *MITF*+ compared to *MITF*− patients; conversely, the multicomponent pattern was more common in *MITF*− than in *MITF*+ patients, as shown in Table 2 (p < 0.001). We could not perform the same analysis on a melanoma-only subset because of the small resulting sample size. However, as regards melanomas, the frequency of global dermoscopic patterns among the two study groups is reported in Table 3. The unspecific pattern was found more frequently in melanomas of *MITF*+ (45% of the lesions) than in those of *MITF*− patients (13% of the lesions), while the multicomponent pattern was seen more frequently among melanomas of *MIT*F− (65% of the lesions) than those of *MITF*+ patients (22%) (Fig. 2). Taking into account that a considerable number of melanocitic lesions belonged to the same patient, we performed again the analysis excluding this outlier patient, to reduce the risk that such a relevant number of non-independent samples could bias our results. Even without the outlier patient, melanocytic lesions in *MITF*+ and *MITF*− patients showed a different distribution of dermoscopic patterns (p = 0.001). Namely, the unspecific was the most frequent dermoscopic pattern found in DN/melanomas of *MITF*+ patients (40%, as opposed to 9% in *MITF*− lesions). Conversely, the multicomponent and the reticular-globular patterns (47% and 28% respectively in *MITF*− lesions) were absent in *MITF*+ lesions.

## Discussion

In our study cohort, the prevalence of the p.E318K germline variant in *CDKN2A*/*CDK4*-negative patients was 2.2%, slightly higher than we previously reported in a smaller series of melanoma patients (1.8%) [13], but in line with Spanish (1.9%) [14], French (2.8%) [6], Australian (3.4–3.6%) [16] and American (2.8%) [12] studies.

Considering that the p.E318K variant is not common in melanoma patients, attempts to determine its effects on *MITF*+ patients’ phenotypical features and cancer predisposition are generally limited by sample size. To our best knowledge, the present study describes the largest cohort of *MITF*+ patients reported to date from a dermoscopic point of view (DN and melanomas), in addition to a genetic, clinical, and pathological perspective.

Concerning the histotype of the first diagnosed melanoma, we validated the association between the p.E318K variant and the nodular subtype previously reported by our group [13]. Indeed, seven out of 22 p.E318K patients (32%) developed a first melanoma with nodular histotype, a significantly higher percentage than the one observed in *MITF*− patients (16%). Our results differ from those of previous studies by other groups, which did not find significant associations of p.E318K with pathological features, possibly due to underpowered study samples [14, 16].

However, Potrony and colleagues reported that during 10 years of dermatological surveillance of patients at high risk of melanoma, the only two fast-growing melanomas (growth rate greater than 0.4 mm per month) were diagnosed in *MITF*+ patients. Of these two lesions, one was a nodular melanoma and the other one was a superficial spreading melanoma (SSM) [14]. However, in our *MITF*+ study group, all nodular melanomas were first diagnosed melanomas, identified during dermatological screening with digital follow-up or clinical examination. Conversely, all subsequent melanomas diagnosed in our *MITF*+ cohort during dermatological follow-up were SSM, and Breslow thickness of melanomas in patients with MPM was always lower than that of the preceding ones, except for one patient, possibly reflecting the intensive dermatological follow-up after the first melanoma diagnosis. However, further investigations with larger series of patients are needed to confirm the association between the p.E318K variant and nodular-type melanoma, and to study the prognostic role of this variant.

Concerning the role of the p.E318K variant in the predisposition to tumors other than melanoma, we confirm the association with renal cell carcinoma (RCC) previously described [6, 7, 13–15]. The association with pancreatic cancer we previously observed in a smaller series of patients [13] was not confirmed here, and therefore remains to be further explored. Although none of our p.E318K patients developed RCC, 18% of them reported a positive family history, as opposed to 4% of *MITF*− patients. Apart from melanoma, the most frequent tumor in *MITF*+ patients was basal cell carcinoma (14% of the patients), in line with previous data reported by Potrony et al. [14].

The finding that *MITF*+ p.E318K was associated with a higher number of histopathologically confirmed DN in our cohort was never reported to date, differently from *CDKN2A* variants, whose possible role in influencing the development of dysplastic melanocytic lesions has already been described [26, 27].

Our study confirms that *MITF*+ patients have an increased risk of developing multiple melanomas and a higher total nevi count compared to *MITF*− patients, as previously reported [6, 7, 13, 14]. Indeed, 28% of *MITF*+ patients in our cohort had more than 50 nevi with > 2 mm diameter, ascompared to 19% of *MITF*− patients. Similarly, previous studies reported high nevi counts in *MITF*+ patients, corroborating the hypothesis that *MITF* is involved in nevogenesis [11, 14, 16].

Of course, as *MITF* p.E318K is considered an intermediate penetrance allele, the possibility that other additional gene’s effects may have affected our results cannot be completely ruled out. However, patients with *CDKN2A* pathogenic variants were excluded from this study, in order to avoid a confounding effect by this gene. Moreover, *MC1R* variants, which influence phototype and are associated with melanoma risk [19, 28], had a similar distribution in the two study groups, therefore not affecting our analyses. *MC1R* RHC variants have also been associated with the likelihood of developing amelanotic/hypomelanotic melanomas [29]. In our cohort, both *MITF*+ patients with amelanotic/hypomelanotic melanomas carried one RHC variants. However, due to the retrospective nature of this study, standardized information on pigmentation was not available, and therefore we could not assess the impact of RHC variants on melanoma pigmentation according to *MITF* germline status.

Although dermoscopic patterns of melanocytic nevi in *MITF*+ and *MITF*− patients have already been reported [11, 14, 16], our study is the first to assess the dermoscopic characterization of DN and melanomas in *MITF*+ patients compared to *MITF*− patients. Previous studies [11, 14, 16] found that the predominant dermoscopic pattern of nevi was the reticular one, both in *MITF*+ and in *MITF*− patients. Moreover, Sturm et al. reported that the frequency of globular nevi was greater in *MITF*+ patients, albeit not significant [16]. In DN and melanomas of our series of *MITF*+ patients, we found 3 prevalent dermoscopic patterns: unspecific, globular-homogeneous and reticular-homogeneous. The unspecific pattern was defined as devoid of structures or with too few structures to identify a pattern, except for the presence of blood vessels. This latter pattern is most frequently found in amelanotic/hypomelanotic melanocytic lesions including amelanotic/hypomelanotic nodular melanomas where it can be associated with polymorphous atypical vessels [30].

While the reticular pattern is suggestive of photoinduced nevogenesis, the globular-homogeneous one, with globules at the periphery of the lesion, expression of lesion growth, suggests that p.E318K variant may also act to force the continuous growth of the nevi/melanomas [31].

Considering only melanomas, the prevalent pattern among the *MITF*+ patients was the unspecific one, a finding that has never been associated with the *MI*TF+ variant to date.

Conversely, the multicomponent pattern was prevalent among the *MITF*− patients, as already reported in the literature [32, 33].

Noteworthy, as a rule, all lesions with unspecific patterns should be biopsied, also in the context of lesions clinically appearing benign, to avoid missing melanomas [23].

Therefore, the detection of this pattern in *MITF*+ patients should alert dermatologist raising the level of suspicion of malignancy.

Since among the 22 *MITF*+ patients one patient developed 10 melanomas (of which 7 dermoscopic images were available), and the different distribution of clinico-pathological-dermoscopic features between the two groups could have been influenced by this single patient, we repeated the analysis excluding this patient. Even though the observed patterns were actually influenced by this patient, the unspecific pattern remained prevalent in *MITF*+ patients and the association remained significant. Dermoscopically, the most common patterns of DN and melanomas (multicomponent, reticular-globular) were almost absent in *MITF*+ patients, while the multicomponent was the most frequent pattern among MITF− patients.

The major limitation in this study is the small number of images included for assessment of dermoscopic pattern in relation to MITF variant which may influence the reliability of these results (only 23 dermoscopic images belonging to four MITF+ patients were available).

## Conclusions

Besides confirming previous results on the association of the p.E318K variant with high number of nevi (> 50 units) and higher risk of melanoma and kidney cancers compared to *MITF*− patients, our study adds the finding that *MITF*+ patients have a higher risk of developing DN than *MITF*− patients. This result underlines the necessity for *MITF*+ patients to follow melanoma prevention programs, including dermatologic surveillance with digital follow-up.

In *MITF*+ patients, any melanocytic lesion with a dermoscopic pattern that digresses from the most commonly dermoscopic patterns reported among the *MITF*− patients, such as multicomponent and reticular-globular patterns, should be examined with caution to avoid missing melanomas that are devoid of structures.

Further studies through an international collaborative effort are crucial to increase the sample size and validate these findings.

## Data Availability

The datasets used and/or analysed during the current study are available from the corresponding author upon request.

## Abbreviations

*ACD*: Adrenocortical dysplasia
AJCC: American Joint Committee on Cancer
*BAP1*: Breast cancer gene 1 (*BRCA1*)-associated protein 1
*CDKN2A*: Cyclin Dependent Kinase Inhibitor 2A
*CDK4*: Cyclin-dependent kinase 4
DN: Dysplastic nevi
*MC1R*: Melanocortin 1 Receptor
*MITF*: Microphthalmia-associated transcription factor
PCR: Polymerase chain reaction
*POT1*: Protection of telomeres 1
RCC: Renal cell carcinoma
*TERF2*: Telomeric repeat-binding factor-2
*TERF2IP*: Telomeric repeat interacting protein
*TERT*: Telomere reverse transcriptase
